# Supplementary material for: Transcriptional coactivation by EHMT2 restricts glucocorticoid-induced insulin resistance in a study with male mice
Source: Nat Commun. 2023 May 30;14:3143. doi: 10.1038/s41467-023-38584-5 (PMC10229547; doi:10.1038/s41467-023-38584-5)
Supplement: Supplementary file 1 — Supplementary Information [file 41467_2023_38584_MOESM1_ESM.pdf]

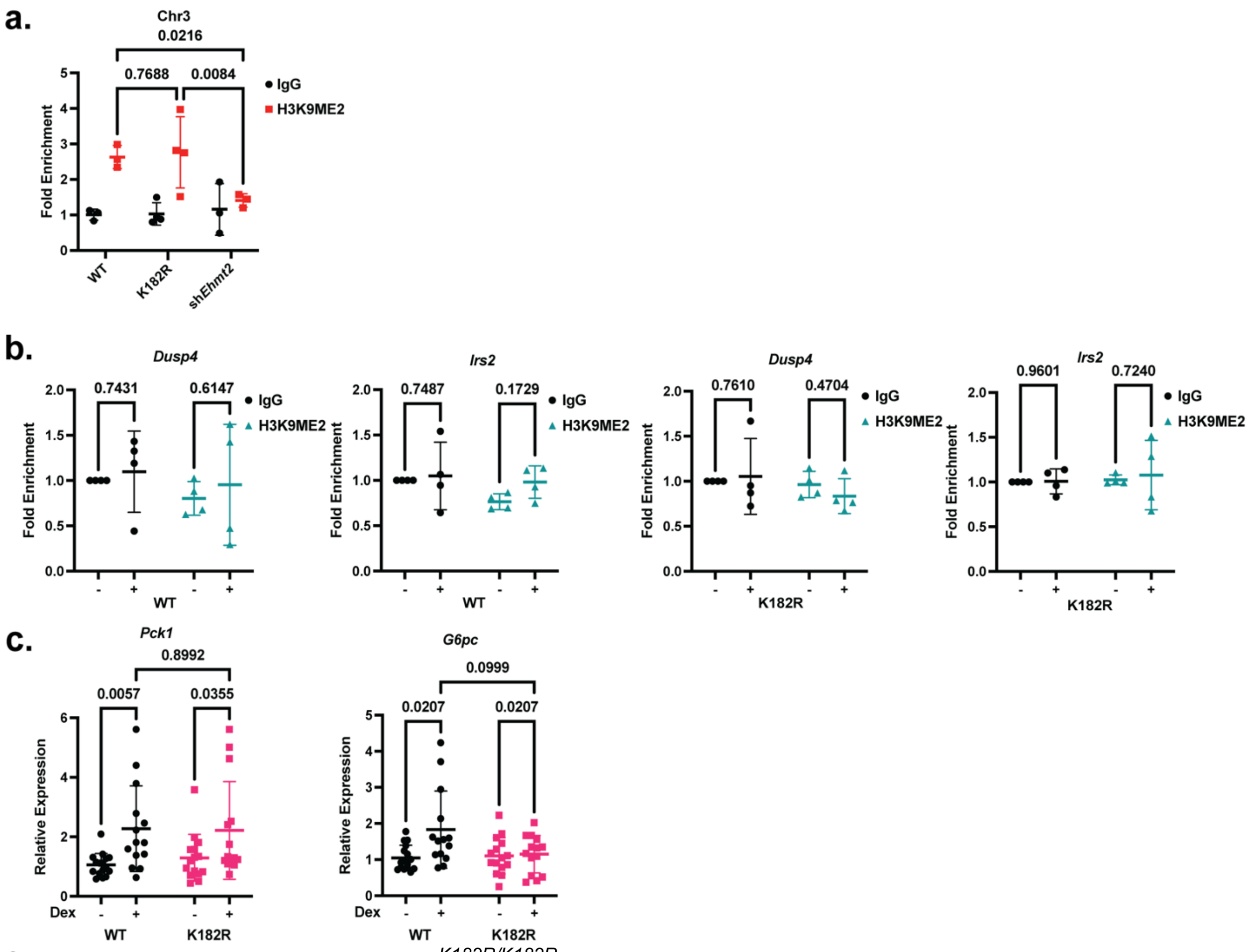

Supplementary Figure 1: *Ehmt2*$^{K182R/K182R}$ mice have similar H3K9ME2 recruitment and gluconeogenic gene expression compared to WT mice(a) ChIP for H3K9ME2 (Red) in mouse liver of WT, *Ehmt2*$^{K182R/K182R}$(K182R), and hepatic shRNA-*Ehmt2* (sh*Ehmt2*) mice (WT n=3, K182R n=4, sh*Ehmt2* n=3 biologically independent mice in 3 independent ChIP experiments) (b) ChIP for H3K9ME2 (Green) in mouse liver of WT and *Ehmt2*$^{K182R/K182R}$ mice treated with Dex in their drinking water for 1 week in *Dusp4* and *Irs2* GBRs n=4 biologically independent mice in 4 independent ChIP experiments (c) Hepatic gluconeogenic gene expression in WT (Black) and *Ehmt2*$^{K182R/K182R}$ (Pink) mice treated with or without Dex for 11 days, (WT- n=16, WT+ n=14, K182R- n=15, K182R+ n=14 biologically independent mice). Statistical tests used were a two-way ANOVA with a Fisher's LSD post-hoc test. The center lines depict the mean and the error bars represent standard deviation. Source data are provided as a Source Data file

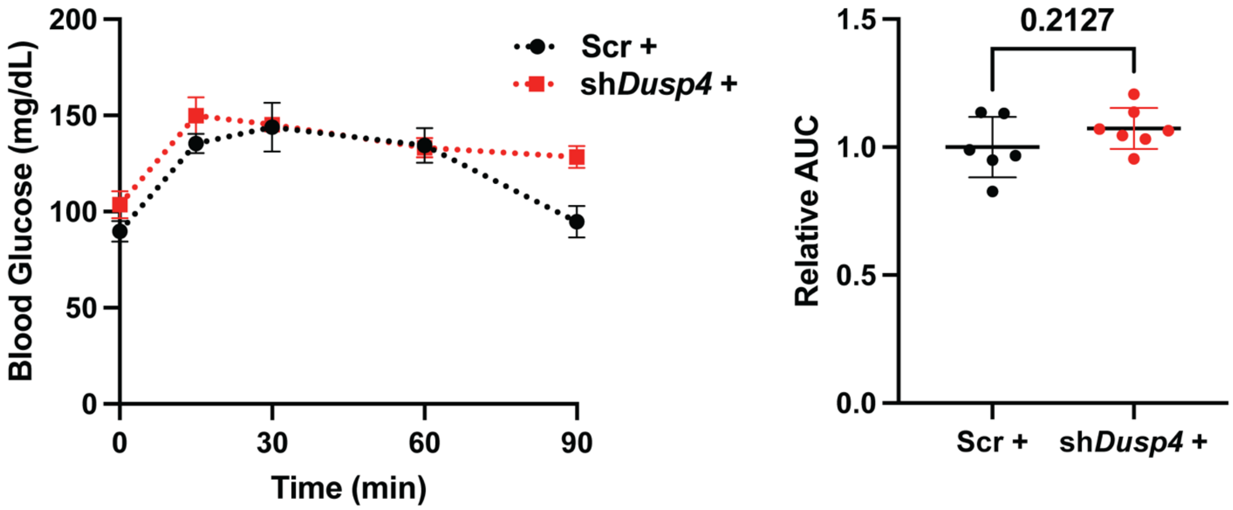

Supplementary Figure 2: Hepatic DUSP4 knockdown does not affect hepatic gluconeogenesis in WT mice. PTT in WT mice with shRNA-Scramble (Black) or *Dusp4* (Red) treated with Dex, (Scr+ n=6, sh*Dusp4*+ n=7 biologically independent mice). Statistical test used was a two-tailed unpaired t test. The center lines depict the mean and the error bars represent SEM for the tolerance test and standard deviation for the relative AUC. Source data are provided as a Source Data file

Supplementary Table 1:

qPCR Primers:		
Primer Name	Forward	Reverse
Rpl19	Tcc ttg gtc tta gac ctg cg	Atg gag cac atc cac aag c
Pck1	Ctg cat aac ggt ctg gac ttc	Cag caa ctg ccc gta ctc c
G6pc	Gac cat aac ata gta tac acc tgc tgc	Gac cat aac ata gta tac acc tgc tgc
ChIP Primers:		
Rpl19	Tcc ttg gtc tta gac ctg cg	Atg gag cac atc cac aag c
Dusp4-(35856151-35856301)GBR	Cac gct aaa ggg aag ggt ga	Tgg gac gca aac cag atg ag
Irs2-(10965648-10965798)GBR	Aga cac tca ggg gaa tgt gc	Atg cag cca ggt aca cca ag
Chr 3-H3k9me2	Ggc tgt ggt ttg gag act cat	Cca tca gct ccc act ggt ta
