# Supplementary material for: Transcriptional coactivation by EHMT2 restricts glucocorticoid-induced insulin resistance in a study with male mice
Source: Nat Commun. 2023 May 30;14:3143. doi: 10.1038/s41467-023-38584-5 (PMC10229547; doi:10.1038/s41467-023-38584-5)
Supplement: Supplementary file 3 — Description of Additional Supplementary Data [file 41467_2023_38584_MOESM3_ESM.pdf]

Description of Additional Supplementary Files

File Name: Supplementary Data 1
Description: html file for RNA-seq

File Name: Supplementary Data 2
Description: the results all genes analyzed in the RNA-seq.
