# Supplementary material for: Transcriptional coactivation by EHMT2 restricts glucocorticoid-induced insulin resistance in a study with male mice
Source: Nat Commun. 2023 May 30;14:3143. doi: 10.1038/s41467-023-38584-5 (PMC10229547; doi:10.1038/s41467-023-38584-5)
Supplement: Supplementary file 4 — Supplementary Data 1 [file 41467_2023_38584_MOESM4_ESM.html]

The effect of K182R on gene expression and regulation by dexamethasone in mouse liver tissue


# The effect of K182R on gene expression and regulation by dexamethasone in mouse liver tissue

#### Miles Pufall

#### 6/3/2021

RNA was isolated from the livers of C57BL/6J mice that are either
wild type or harbor a homozygous K182R mutation in EMHT2 treated with
either vehicle or dexamethasone for 5 hours. The goal was to identify
genes whose regulation in response to dex changes due to the mutation.
Three biological repeats of each condition were performed, for a total
of 12 samples.

## A. Processing

### A.1 Trim adapters off the reads using TrimGalore!

```
trim_galore -o Sample-2 Sample-2/Sample-2_1.fq.gz`
```

This probably isn’t 100% necessary, as the reads are single and
short, but did it anyway.

### A.2 Quantifying counts

I’ll use Salmon (v 1.3.0) to make count table (https://combine-lab.github.io/salmon/)

Downloaded transcriptome in fasta format from ensembl (ftp://ftp.ensembl.org/pub/release-101/fasta/mus\_musculus/cdna/Mus\_musculus.GRCm38.cdna.all.fa.gz)

As well as the gene structures in GTF format (ftp://ftp.ensembl.org/pub/release-101/gtf/mus\_musculus/Mus\_musculus.GRCm38.101.gtf.gz)

The first step - index the transcriptome:

```
salmon index -t Mus_musculus.GRCm38.cdna.all.fa -i GRCm38_index
```

The map the reads:

```
#!/bin/bash
for fn in Sample-{1..12};
do
samp=`basename ${fn}`
echo "Processing sample ${samp}"
salmon quant -i GRCm38_index  -l A \
        -r ${samp}/${samp}_1_trimmed.fq.gz \
        -p 4 --validateMappings --seqBias --gcBias --numBootstraps 50 -o quants/${samp}_quant
done
```

### A.3 Import data into R

We’ll use tximeta.

```
list.files(file.path("quants"))
```

```
##  [1] "Sample-1_quant"  "Sample-10_quant" "Sample-11_quant" "Sample-12_quant"
##  [5] "Sample-2_quant"  "Sample-3_quant"  "Sample-4_quant"  "Sample-5_quant" 
##  [9] "Sample-6_quant"  "Sample-7_quant"  "Sample-8_quant"  "Sample-9_quant"
```

```
files <- file.path("quants", paste0("Sample-", 1:12, "_quant"), "quant.sf")
names <- paste0("Sample_", 1:12)
cell <- factor(c("wt", "wt", "wt", "wt", "wt", "wt", "K182R", "K182R", "K182R", "K182R", "K182R", "K182R"), levels = c("wt","K182R"))
treat <- factor(c("PBS", "PBS", "PBS", "dex", "dex", "dex", "PBS", "PBS", "PBS", "dex", "dex", "dex"), levels = c("PBS", "dex"))
coldata <- tibble(files, names, cell, treat)
file.exists(coldata$files)
```

```
##  [1] TRUE TRUE TRUE TRUE TRUE TRUE TRUE TRUE TRUE TRUE TRUE TRUE
```

```
se <- tximeta(coldata)
```

```
## importing quantifications
```

```
## reading in files with read_tsv
```

```
## 1 2 3 4 5 6 7 8 9 10 11 12 
## found matching transcriptome:
## [ Ensembl - Mus musculus - release 102 ]
## loading existing EnsDb created: 2022-11-21 05:09:02
## loading existing transcript ranges created: 2022-11-21 05:09:04
```

```
gse <- summarizeToGene(se)
```

```
## loading existing EnsDb created: 2022-11-21 05:09:02
## obtaining transcript-to-gene mapping from database
## loading existing gene ranges created: 2022-11-21 05:09:07
## summarizing abundance
## summarizing counts
## summarizing length
## summarizing inferential replicates
```

Make count table for deposit into GEO

```
count_table <- assays(gse)$counts
colnames(count_table) <- paste0(coldata$cell, "_", coldata$treat)
count_table_df <- as.data.frame(count_table)

write.csv(count_table_df, "k182r_count_table.csv")
```

### A.4 How many reads mapped per sample?

```
round(colSums(assay(gse)) / 1e6, 1 )
```

```
##  Sample_1  Sample_2  Sample_3  Sample_4  Sample_5  Sample_6  Sample_7  Sample_8 
##      20.4      20.4      20.5      20.4      20.0      20.0      20.5      20.3 
##  Sample_9 Sample_10 Sample_11 Sample_12 
##      20.6      20.1      20.0      20.1
```

That’s incredibly consistent.

Make a DESeq object

```
dds <- DESeqDataSet(gse, design = ~ cell + treat + cell:treat)
```

```
## using counts and average transcript lengths from tximeta
```

The model here is important - it specifies that both treatment and
mutational status (cell) contribute to expression, but also looks for
how the combination of these two factors (cell:treat) interact. We are
particularly interested in the genes that do not adhere to the linear
combination of mutation and treatment, meaning that mutation has an
unexpected large effect on gene expression in response to dex, thus the
interaction term is key.

Filter out low/non expressed genes by getting rids of genes with <
2 counts per gen on average. Kind of aggressive.

```
keep <- rowSums(counts(dds)) > 24
dds <- dds[keep,]
nrow(dds)
```

```
## [1] 15504
```

Transform the data to visualize

```
vsd <- vst(dds, blind = FALSE)
```

```
## using 'avgTxLength' from assays(dds), correcting for library size
```

## B. Exploratory Data Analysis

### B.1 Do the samples cluster based on mutation or treatment?

```
sampleDists <- dist(t(assay(vsd)))

sampleDistMatrix <- as.matrix( sampleDists )

rownames(sampleDistMatrix) <- paste(vsd$cell, vsd$treat, sep = " - " )

colnames(sampleDistMatrix) <- NULL

colors <- colorRampPalette( rev(brewer.pal(9, "Blues")) )(255)

pheatmap(sampleDistMatrix,
         clustering_distance_rows = sampleDists,
         clustering_distance_cols = sampleDists,
         col = colors)
```

```
poisd <- PoissonDistance(t(counts(dds)))
samplePoisDistMatrix <- as.matrix( poisd$dd )
rownames(samplePoisDistMatrix) <- paste( dds$cell, dds$treat, sep=" - " )
colnames(samplePoisDistMatrix) <- NULL
pheatmap(samplePoisDistMatrix,
         clustering_distance_rows = poisd$dd,
         clustering_distance_cols = poisd$dd,
         col = colors)
```

### B.2 PCA plot

```
plotPCA(vsd, intgroup = c("treat", "cell"))
```

This shows that the most prominent source of variation is treatment
with dex. That’s good. The next biggest source of variation is mutation
status - which is also good. There is one outlier in the PBS:K182R group
- we’ll see if that has an effect on differential expression
significance.

## C. Differential Expression Analysis

```
dds <- DESeq(dds)
```

```
## estimating size factors
```

```
## using 'avgTxLength' from assays(dds), correcting for library size
```

```
## estimating dispersions
```

```
## gene-wise dispersion estimates
```

```
## mean-dispersion relationship
```

```
## final dispersion estimates
```

```
## fitting model and testing
```

```
res <- results(dds)
resultsNames(dds)
```

```
## [1] "Intercept"          "cell_K182R_vs_wt"   "treat_dex_vs_PBS"  
## [4] "cellK182R.treatdex"
```

### C.1 Gene lists

#### C.1.a How many genes changes expression in G9A K182R mutant?

K182R vs WT

```
mut_res <- results(dds, name = "cell_K182R_vs_wt", alpha = 0.01)
summary(mut_res)
```

```
## 
## out of 15504 with nonzero total read count
## adjusted p-value < 0.01
## LFC > 0 (up)       : 23, 0.15%
## LFC < 0 (down)     : 31, 0.2%
## outliers [1]       : 53, 0.34%
## low counts [2]     : 2093, 13%
## (mean count < 8)
## [1] see 'cooksCutoff' argument of ?results
## [2] see 'independentFiltering' argument of ?results
```

```
plotMA(mut_res, ylim = c(-10,10))
```

#### C.1.b How many genes are regulated by dex in WT mice?

WT + dex

```
wt_dex_res <- results(dds, contrast=c("treat","dex","PBS"), alpha = 0.01)
head(wt_dex_res[ order(wt_dex_res$padj, decreasing = FALSE), ], 10)
```

```
## log2 fold change (MLE): treat dex vs PBS 
## Wald test p-value: treat dex vs PBS 
## DataFrame with 10 rows and 6 columns
##                      baseMean log2FoldChange     lfcSE      stat      pvalue
##                     <numeric>      <numeric> <numeric> <numeric>   <numeric>
## ENSMUSG00000071350  1149.4053        3.30512  0.189076   17.4803 2.02385e-68
## ENSMUSG00000066361   342.9636        3.13408  0.191481   16.3676 3.25846e-60
## ENSMUSG00000015312   490.8664        5.33583  0.326701   16.3325 5.79925e-60
## ENSMUSG00000026131  2811.3207        1.85510  0.123240   15.0527 3.31326e-51
## ENSMUSG00000056313   911.1088        2.98650  0.222968   13.3943 6.52683e-41
## ENSMUSG00000032092  1463.4588        1.47126  0.113870   12.9205 3.44694e-38
## ENSMUSG00000079012 13987.9438        2.81229  0.219224   12.8284 1.13670e-37
## ENSMUSG00000039956  3058.1703        2.67526  0.213866   12.5090 6.66177e-36
## ENSMUSG00000021981  3089.4072        2.15297  0.173431   12.4140 2.19353e-35
## ENSMUSG00000111259    70.0657        3.90011  0.322405   12.0969 1.09635e-33
##                           padj
##                      <numeric>
## ENSMUSG00000071350 2.76377e-64
## ENSMUSG00000066361 2.22487e-56
## ENSMUSG00000015312 2.63982e-56
## ENSMUSG00000026131 1.13115e-47
## ENSMUSG00000056313 1.78261e-37
## ENSMUSG00000032092 7.84523e-35
## ENSMUSG00000079012 2.21754e-34
## ENSMUSG00000039956 1.13716e-32
## ENSMUSG00000021981 3.32832e-32
## ENSMUSG00000111259 1.49717e-30
```

```
summary(wt_dex_res)
```

```
## 
## out of 15504 with nonzero total read count
## adjusted p-value < 0.01
## LFC > 0 (up)       : 677, 4.4%
## LFC < 0 (down)     : 545, 3.5%
## outliers [1]       : 53, 0.34%
## low counts [2]     : 1795, 12%
## (mean count < 6)
## [1] see 'cooksCutoff' argument of ?results
## [2] see 'independentFiltering' argument of ?results
```

```
plotMA(wt_dex_res, ylim = c(-10,10))
```

```
updown_tbl <- wt_dex_res %>%
  as.data.frame() %>%
  mutate(reg = case_when(padj < 0.01 & log2FoldChange > 0 ~ "Up",  padj < 0.01 & log2FoldChange < 0 ~ "Down")) %>%
  drop_na() %>%
  count(reg) %>%
  rename(WT = n)
```

#### C.1.c How many genes are regulated by dex in mutant background?

K182R + dex

```
k182r_dex_res <- results(dds, list(c("treat_dex_vs_PBS","cellK182R.treatdex")), alpha = 0.01)

summary(k182r_dex_res)
```

```
## 
## out of 15504 with nonzero total read count
## adjusted p-value < 0.01
## LFC > 0 (up)       : 1009, 6.5%
## LFC < 0 (down)     : 819, 5.3%
## outliers [1]       : 53, 0.34%
## low counts [2]     : 1795, 12%
## (mean count < 6)
## [1] see 'cooksCutoff' argument of ?results
## [2] see 'independentFiltering' argument of ?results
```

```
plotMA(k182r_dex_res, ylim = c(-10,10))
```

```
updown_mut_tbl <- k182r_dex_res %>%
  as.data.frame() %>%
  mutate(reg = case_when(padj < 0.01 & log2FoldChange > 0 ~ "Up",  padj < 0.01 & log2FoldChange < 0 ~ "Down")) %>%
  drop_na() %>%
  count(reg) %>%
  rename(K182R = n)
```

---

## Figure S1: Genes up and downregulated by dexamethasone in EHMT2-WT and -K182R mutant

---

```
updown_tbl %>%
  left_join(updown_mut_tbl, by = "reg") %>%
  mutate(`WT%` = round(WT/sum(.$WT)*100, 0)) %>%
  mutate(`K182R%` = round(K182R/sum(.$K182R)*100, 0)) %>%
  pivot_longer(cols = WT:K182R, names_to = "mutant", values_to = "values") %>%
  ggplot() +
  geom_col(aes(mutant, values, fill = reg), width = 0.7, position = position_dodge(0.8)) +
  labs(y = "Number of Genes", x = NULL) +
  scale_fill_manual(values = c("red", "blue"), name = "Regulation") +
  theme_bw() +
  theme(axis.title = element_text(face = "bold", size = 14), axis.text=element_text(face = "bold", size=11), legend.position = c(0.85,0.9), legend.text = element_text(size = 11), legend.title = element_text(face = "bold", size = 12))
```

Figure S1: Genes up and downregulated by dexamethasone in EHMT2-WT and
-K182R mutant

#### D.1.d How many genes change in regulation due to the mutation?

```
res_int <- results(dds, name="cellK182R.treatdex", alpha = 0.01)

plotMA(res_int, ylim  = c(-10, 10))
```

```
summary(res_int)
```

```
## 
## out of 15504 with nonzero total read count
## adjusted p-value < 0.01
## LFC > 0 (up)       : 5, 0.032%
## LFC < 0 (down)     : 2, 0.013%
## outliers [1]       : 53, 0.34%
## low counts [2]     : 0, 0%
## (mean count < 2)
## [1] see 'cooksCutoff' argument of ?results
## [2] see 'independentFiltering' argument of ?results
```

At first blush this looks odd - the K182R mutant appears to regulate
more genes. Let’s use a grouping model and make sure we are looking at
the right things.

```
dds$group <- factor(paste0(dds$cell, dds$treat))
design(dds) <- ~ group
dds <- DESeq(dds)
```

```
## using pre-existing normalization factors
```

```
## estimating dispersions
```

```
## found already estimated dispersions, replacing these
```

```
## gene-wise dispersion estimates
```

```
## mean-dispersion relationship
```

```
## final dispersion estimates
```

```
## fitting model and testing
```

```
resultsNames(dds)
```

```
## [1] "Intercept"                  "group_K182RPBS_vs_K182Rdex"
## [3] "group_wtdex_vs_K182Rdex"    "group_wtPBS_vs_K182Rdex"
```

Results for dex in wt cells

```
wt_dex_grp <- results(dds, contrast = c("group", "wtdex", "wtPBS"), alpha = 0.01)

summary(wt_dex_grp)
```

```
## 
## out of 15504 with nonzero total read count
## adjusted p-value < 0.01
## LFC > 0 (up)       : 676, 4.4%
## LFC < 0 (down)     : 544, 3.5%
## outliers [1]       : 53, 0.34%
## low counts [2]     : 1795, 12%
## (mean count < 6)
## [1] see 'cooksCutoff' argument of ?results
## [2] see 'independentFiltering' argument of ?results
```

```
plotMA(wt_dex_grp, ylim = c(-10,10))
```

Looks perfect, actually, so no worries.

#### D.1.e How many genes have different expression after dex treatment in the mutant?

```
mut_dex <- results(dds, contrast = c("group", "K182Rdex", "wtdex"), alpha = 0.01)

summary(mut_dex)
```

```
## 
## out of 15504 with nonzero total read count
## adjusted p-value < 0.01
## LFC > 0 (up)       : 13, 0.084%
## LFC < 0 (down)     : 8, 0.052%
## outliers [1]       : 53, 0.34%
## low counts [2]     : 2990, 19%
## (mean count < 13)
## [1] see 'cooksCutoff' argument of ?results
## [2] see 'independentFiltering' argument of ?results
```

```
plotMA(mut_dex, ylim = c(-10,10))
```

#### Make some tables with gene names

##### Function to add gene symbols

```
add_geneidsq <- function(genelist) {
  genelist$symbol <- mapIds(org.Mm.eg.db, keys=row.names(genelist), column="SYMBOL", keytype="ENSEMBL", multiVals="first")
  genelist$entrez <- mapIds(org.Mm.eg.db, keys=row.names(genelist), column="ENTREZID", keytype="ENSEMBL", multiVals="first")
  genelist$genename <- mapIds(org.Mm.eg.db, keys=row.names(genelist), column="GENENAME", keytype="ENSEMBL", multiVals="first")
  return(genelist)
}
```

##### Add gene names

```
#Convert them into tables

mut_drg <- mut_res %>%
  as.data.frame()
names(mut_drg) <- paste0("mut.", names(mut_drg))
mut_drg <- mut_drg %>%
  rownames_to_column(var = "ensembl")

wt_dex_drg <- wt_dex_res %>%
  as.data.frame()
names(wt_dex_drg) <- paste0("wt_dex.", names(wt_dex_drg))
wt_dex_drg <- wt_dex_drg %>%
  rownames_to_column(var = "ensembl")

k182r_dex_drg <- k182r_dex_res %>%
  as.data.frame()
names(k182r_dex_drg) <- paste0("k182r_dex.", names(k182r_dex_drg))
k182r_dex_drg <- k182r_dex_drg %>%
  rownames_to_column(var = "ensembl")

int_drg <- res_int %>%
  as.data.frame()
names(int_drg) <- paste0("int.", names(int_drg))
int_drg <- int_drg %>%
  rownames_to_column(var = "ensembl")

mut_dex_deg <- mut_dex %>%
  as.data.frame()
names(mut_dex_deg) <- paste0("md.", names(mut_dex_deg))
mut_dex_deg <- mut_dex_deg %>%
  rownames_to_column(var = "ensembl")
```

##### Then turn them into tables and merge them

```
drg_merge <- wt_dex_drg %>%
  left_join(k182r_dex_drg, by = "ensembl") %>%
  left_join(mut_drg, by = "ensembl") %>%
  left_join(int_drg, by = "ensembl") %>%
  left_join(mut_dex_deg, by = "ensembl") %>%
  dplyr::filter(wt_dex.baseMean > 30)

## then annotate and get rid of genes w/ no log2foldchange
add_geneids_tbl <- function(genelist) {
  genelist$symbol <- mapIds(org.Mm.eg.db, keys=genelist$ensembl, column="SYMBOL", keytype="ENSEMBL", multiVals="first")
  genelist$entrez <- mapIds(org.Mm.eg.db, keys=genelist$ensembl, column="ENTREZID", keytype="ENSEMBL", multiVals="first")
  genelist$genename <- mapIds(org.Mm.eg.db, keys=genelist$ensembl, column="GENENAME", keytype="ENSEMBL", multiVals="first")
  #genelist <- genelist %>% drop_na(log2FoldChange)
  return(genelist)
}
  
drg_merge_tbl <- add_geneids_tbl(drg_merge)
```

```
## 'select()' returned 1:many mapping between keys and columns
## 'select()' returned 1:many mapping between keys and columns
## 'select()' returned 1:many mapping between keys and columns
```

```
write_csv(drg_merge_tbl, "G9A_k182r_full_gene_reg_table.csv")
```

The table of full results is
`G9A_k182r_full_gene_reg_table.csv`

### D.2 Does mutation of K182R cause a *general* difference in how dex regulates genes?

---

## Figure S2: Most genes in the K182R mutant are regulated similarly to WT in response to Dex

---

```
#Filter for genes that are regulated by dex in either wt or K182R background
drg <- dplyr::filter(drg_merge_tbl, k182r_dex.log2FoldChange < 20 & wt_dex.log2FoldChange < 20)

drg %>%
  dplyr::filter(wt_dex.padj <= 0.05 | k182r_dex.padj <= 0.05) %>%
  ggscatter(x = "wt_dex.log2FoldChange", y = "k182r_dex.log2FoldChange",
            add = "reg.line", conf.int = TRUE,
            cor.coef = TRUE, cor.method = "pearson", 
            xlab = "WT + Dex, log2 Fold Change", ylab = "K182R + Dex, log2 Fold Change") +
  xlim(-10, 10) + ylim(-12, 12) +
  geom_abline(intercept = 0, slope = 1, colour = "red")
```

```
## `geom_smooth()` using formula = 'y ~ x'
```

Figure S1: The K182R mutation does not cause an overall effect on dex
regulation fo gene expression

This would seem to say no. There are individual genes that are
differently regulated - and in fact go from largely unregulated in WT to
strongly regulated in the mutant.

### D.3 Genes that change expression after treatment with dex in K182R

```
#Convert to long table
long <- drg_merge_tbl %>%
  pivot_longer(wt_dex.baseMean:md.padj, names_to = c("treatment",".value"), names_pattern = "(^\\w+).(\\w+$)") %>%
  mutate(treatment = factor(treatment, levels = c("mut", "md","wt_dex", "k182r_dex", "int")))

base_delta <- drg_merge_tbl %>%
  dplyr::filter(mut.padj < 0.01) %>%
  dplyr::select(symbol) %>%
  dplyr::filter(!is.na(symbol)) %>%
  pull(symbol)
#  drop_na()

long_filt <- dplyr::filter(long, log2FoldChange < 15 & log2FoldChange > -15 & symbol != "Ppp1ccb")

long_filt %>%
  dplyr::filter(symbol %in% base_delta) %>%
  ggplot(aes(treatment, log2FoldChange, fill = treatment)) + 
  labs(x = "", y = "log2FoldChange") + ggtitle("Genes that change expression in K182R w/o dex") +
  geom_col(position = "dodge") +
  scale_fill_viridis(discrete = T, option = "E") +
  facet_wrap(~symbol) +theme_bw() + 
  theme(legend.position="none", axis.text.x = element_text(angle = 45, hjust = 1)) +
  geom_errorbar(aes(ymin=log2FoldChange-lfcSE, ymax=log2FoldChange+lfcSE), position = position_dodge(width = 0.9), width=0.5, colour="black", size = 0.5)
```

```
## Warning: Using `size` aesthetic for lines was deprecated in ggplot2 3.4.0.
## ℹ Please use `linewidth` instead.
```

**Comparison labels**  
**mut** = K182R vs. wt (vehicle)  
**md** = K182R vs. wt (after dex treatment)  
**wt\_dex** = wt + dex vs. wt + vehicle  
**k182r\_dex** = K182R + dex vs. K182R + vehicle  
**int** = (K182R + dex vs. K182R + vehicle) - (wt + dex
vs. wt + vehicle)

### D.4 Genes that are upregulated in wild-type, but not as up-regulated in K182R

```
worse_K182R <- drg_merge_tbl %>%
  dplyr::filter(wt_dex.padj < 0.01 & wt_dex.log2FoldChange > 0) %>% 
  dplyr::filter(k182r_dex.log2FoldChange < wt_dex.log2FoldChange) %>%
  dplyr::filter(!is.na(symbol))

## Make a table for these genes
write_csv(worse_K182R, "k182r_act_impaired.csv")

## The *most* impaired in activation upon mutation
worst_K182R <- drg_merge_tbl %>%
  dplyr::filter(wt_dex.padj < 0.01 & wt_dex.log2FoldChange > 0) %>% 
  dplyr::filter(k182r_dex.log2FoldChange < 0.5*wt_dex.log2FoldChange)  %>%
  dplyr::select(symbol) %>%
  dplyr::filter(!is.na(symbol)) %>%
  pull(symbol)
```

This gene list is `k182r_act_impaired.csv`

```
long_filt %>%
  dplyr::filter(symbol %in% worst_K182R) %>%
  ggplot(aes(treatment, log2FoldChange, fill = treatment)) + 
  labs(x = "", y = "log2FoldChange") + ggtitle("Most Activation Impaired Genes in K182R (>2 fold impaired)") +
  geom_col(position = "dodge") +
  scale_fill_viridis(discrete = T, option = "E") +
  facet_wrap(~symbol) +theme_bw() + 
  theme(legend.position="none", axis.text.x = element_text(angle = 45, hjust = 1)) +
  geom_errorbar(aes(ymin=log2FoldChange-lfcSE, ymax=log2FoldChange+lfcSE), position = position_dodge(width = 0.9), width=0.5, colour="black", size = 0.5)
```

Among these, Dusp4 and Irs2 are related to glucose metabolism and
insulin sensitivity.

**Comparison labels**  
**mut** = K182R vs. wt (vehicle)  
**md** = K182R vs. wt (after dex treatment)  
**wt\_dex** = wt + dex vs. wt + vehicle  
**k182r\_dex** = K182R + dex vs. K182R + vehicle  
**int** = (K182R + dex vs. K182R + vehicle) - (wt + dex
vs. wt + vehicle)

---

### Figure 4C: Effect of K182R on dex regulation of Dusp4 and Irs2

---

```
long_filt %>%
  dplyr::filter(symbol %in% c("Dusp4", "Irs2")) %>%
  dplyr::filter(treatment %in% c("mut", "wt_dex", "k182r_dex")) %>%
  ggplot(aes(treatment, log2FoldChange, fill = treatment)) + 
  labs(x = "", y = "log2FoldChange") +
  geom_col(position = "dodge") +
  scale_fill_viridis(discrete = T, option = "E") +
  scale_x_discrete(labels=c("mut" = "K182R vs WT","wt_dex" = "WT + Dex", "mut_dex" = "K182R + Dex")) +
  facet_wrap(~symbol) +
  theme_bw() + 
  theme(axis.title = element_text(face = "bold", size = 14), axis.text=element_text(face = "bold", size=11), legend.position="none", axis.text.x = element_text(angle = 45, hjust = 1), strip.text.x = element_text(size = 15, color = "white", face = "bold"), strip.background = element_rect(
     color="black", fill="black", size=1.2, linetype="solid")) +
  geom_errorbar(aes(ymin=log2FoldChange-lfcSE, ymax=log2FoldChange+lfcSE), position = position_dodge(width = 0.8), width=0.25, colour="black", size = 0.5)
```

```
## Warning: The `size` argument of `element_rect()` is deprecated as of ggplot2 3.4.0.
## ℹ Please use the `linewidth` argument instead.
```

```
ggsave("dusp4_irs2_exp.pdf", width = 4, height = 8, units = "in")

long_filt %>%
  dplyr::filter(symbol %in% c("Pck1", "G6pc")) %>%
  dplyr::filter(treatment %in% c("mut", "wt_dex", "k182r_dex")) %>%
  ggplot(aes(treatment, log2FoldChange, fill = treatment)) + 
  labs(x = "", y = "log2FoldChange") +
  geom_col(position = "dodge") +
  scale_fill_viridis(discrete = T, option = "E") +
  scale_x_discrete(labels=c("mut" = "K182R vs WT","wt_dex" = "WT + Dex", "mut_dex" = "K182R + Dex")) +
  facet_wrap(~symbol) +
  theme_bw() + 
  theme(axis.title = element_text(face = "bold", size = 14), axis.text=element_text(face = "bold", size=11), legend.position="none", axis.text.x = element_text(angle = 45, hjust = 1), strip.text.x = element_text(size = 15, color = "white", face = "bold"), strip.background = element_rect(
     color="black", fill="black", size=1.2, linetype="solid")) +
  geom_errorbar(aes(ymin=log2FoldChange-lfcSE, ymax=log2FoldChange+lfcSE), position = position_dodge(width = 0.8), width=0.25, colour="black", size = 0.5)
```

```
ggsave("pck1_g6pc_exp.pdf", width = 4, height = 8, units = "in")

long_filt %>%
  dplyr::filter(symbol %in% c("Pck1", "G6pc")) %>%
  dplyr::filter(treatment %in% c("mut", "wt_dex", "k182r_dex")) %>%
  ggplot(aes(treatment, log2FoldChange, fill = treatment)) + 
  labs(x = "", y = "log2FoldChange") +
  geom_col(position = "dodge") +
  scale_fill_viridis(discrete = T, option = "E") +
  scale_x_discrete(labels=c("mut" = "K182R vs WT","wt_dex" = "WT + Dex", "mut_dex" = "K182R + Dex")) +
  facet_wrap(~symbol) +
  theme_bw() + 
  theme(axis.title = element_text(face = "bold", size = 14), axis.text=element_text(face = "bold", size=11), legend.position="none", axis.text.x = element_text(angle = 45, hjust = 1), strip.text.x = element_text(size = 15, color = "white", face = "bold"), strip.background = element_rect(
     color="black", fill="black", size=1.2, linetype="solid")) +
  geom_errorbar(aes(ymin=log2FoldChange-lfcSE, ymax=log2FoldChange+lfcSE), position = position_dodge(width = 0.8), width=0.25, colour="black", size = 0.5)
```

```
ggsave("pck1_g6pc_exp.pdf", width = 4, height = 8, units = "in")
```

### D.5 Genes whose regulation by dex is enhanced in K182R

```
better_K182R <- drg_merge_tbl %>%
  dplyr::filter(wt_dex.padj < 0.01 | k182r_dex.padj < 0.01) %>% 
  dplyr::filter((wt_dex.log2FoldChange > 0 & k182r_dex.log2FoldChange > wt_dex.log2FoldChange) | (wt_dex.log2FoldChange < 0 & k182r_dex.log2FoldChange < wt_dex.log2FoldChange)) %>%
  dplyr::filter(!is.na(symbol))

## Table
write_csv(better_K182R, "enhanced_reg_k182r.csv")

## Strongest - > 2 fold more strongly regulated

best_K182R <- drg_merge_tbl %>%
  dplyr::filter(wt_dex.padj < 0.01 | k182r_dex.padj < 0.01) %>% 
  dplyr::filter((wt_dex.log2FoldChange > 0 & 0.5*k182r_dex.log2FoldChange > wt_dex.log2FoldChange) | (wt_dex.log2FoldChange < 0 & 0.5*k182r_dex.log2FoldChange < wt_dex.log2FoldChange)) %>%
  dplyr::filter(!is.na(symbol))

write_csv(best_K182R, "best_enhanced_reg_k182r.csv")
```

The enhanced gene list is `enhanced_reg_k182r.csv` and
most enhanced are `best_enhanced_reg_k182r.csv`

This ends up being a lot of genes

```
## Upregulated genes
tally(best_K182R, wt_dex.log2FoldChange > 0)
```

```
##     n
## 1 184
```

```
tally(best_K182R, wt_dex.log2FoldChange < 0)
```

```
##     n
## 1 174
```

### D.6 Genes newly regulated in K182R

We want ones that are really not regulated in wt (adjp > 0.1) and
for sure regulated in K182R (adjp < 0.01)

```
new_K182R <- drg_merge_tbl %>%
  dplyr::filter(wt_dex.padj > 0.1 & k182r_dex.padj < 0.01) %>% 
  dplyr::filter(!is.na(symbol))

write_csv(new_K182R, "newly_regulated_K182R.csv")

## the strongest of these

strong_new_K182R <- drg_merge_tbl %>%
  dplyr::filter(wt_dex.padj > 0.1 & k182r_dex.padj < 0.01) %>% 
  dplyr::filter((k182r_dex.log2FoldChange > 0 & (k182r_dex.log2FoldChange - wt_dex.log2FoldChange) > 1) | (k182r_dex.log2FoldChange < 0 & (k182r_dex.log2FoldChange - wt_dex.log2FoldChange) < -1)) %>%
  dplyr::filter(!is.na(symbol))

write_csv(strong_new_K182R, "strong_new_reg_K182R.csv")


strong <- pull(strong_new_K182R, symbol)

long_filt %>%
  dplyr::filter(symbol %in% strong) %>%
  dplyr::filter(treatment == "wt_dex" | treatment == "k182r_dex") %>%
  ggplot(aes(treatment, log2FoldChange, fill = treatment)) + 
  labs(x = "", y = "log2FoldChange") + ggtitle("Genes regulated strongly in K182R only") +
  geom_col(position = "dodge") +
  scale_fill_viridis(discrete = T, option = "E") +
  facet_wrap(~symbol) +theme_bw() + 
  theme(legend.position="none", axis.text.x = element_text(angle = 45, hjust = 1)) +
  geom_errorbar(aes(ymin=log2FoldChange-lfcSE, ymax=log2FoldChange+lfcSE), position = position_dodge(width = 0.9), width=0.5, colour="black", size = 0.5)
```

Well, there are 440 newly regulated in K182R and in 60 of these the
effect is very strong (> 2 fold difference in regulation)

**Comparison labels**  
**wt\_dex** = wt + dex vs. wt + vehicle  
**k182r\_dex** = K182R + dex vs. K182R + vehicle

Newly regulated genes in G9A K182R are in table
`newly_regulated_K182R.csv` with the strongest ones
`strong_new_reg_K182R.csv`

Repress in wt, then lose repression or activation

```
repress_fail_K182R <- drg_merge_tbl %>%
  dplyr::filter(wt_dex.padj < 0.01 & wt_dex.log2FoldChange < 0 & k182r_dex.pvalue > 0.1) %>% 
  dplyr::filter(!is.na(symbol))

write_csv(repress_fail_K182R, "repress_fail_K182R.csv")

## the strongest of these

rf_symbol <- pull(repress_fail_K182R, symbol)

long_filt %>%
  dplyr::filter(symbol %in% rf_symbol) %>%
  dplyr::filter(treatment == "wt_dex" | treatment == "k182r_dex") %>%
  ggplot(aes(treatment, log2FoldChange, fill = treatment)) + 
  labs(x = "", y = "log2FoldChange") + ggtitle("Genes that fail to be repressed in K182R mutant") +
  geom_col(position = "dodge") +
  scale_fill_viridis(discrete = T, option = "E") +
  facet_wrap(~symbol) +theme_bw() + 
  theme(legend.position="none", axis.text.x = element_text(angle = 45, hjust = 1)) +
  geom_errorbar(aes(ymin=log2FoldChange-lfcSE, ymax=log2FoldChange+lfcSE), position = position_dodge(width = 0.9), width=0.5, colour="black", size = 0.5)
```

**Comparison labels**  
**wt\_dex** = wt + dex vs. wt + vehicle  
**k182r\_dex** = K182R + dex vs. K182R + vehicle

```
sessionInfo()
```

```
## R version 4.2.2 (2022-10-31)
## Platform: x86_64-apple-darwin17.0 (64-bit)
## Running under: macOS Big Sur ... 10.16
## 
## Matrix products: default
## BLAS:   /Library/Frameworks/R.framework/Versions/4.2/Resources/lib/libRblas.0.dylib
## LAPACK: /Library/Frameworks/R.framework/Versions/4.2/Resources/lib/libRlapack.dylib
## 
## locale:
## [1] en_US.UTF-8/en_US.UTF-8/en_US.UTF-8/C/en_US.UTF-8/en_US.UTF-8
## 
## attached base packages:
## [1] stats4    stats     graphics  grDevices utils     datasets  methods  
## [8] base     
## 
## other attached packages:
##  [1] viridis_0.6.2               viridisLite_0.4.1          
##  [3] gridExtra_2.3               ggpubr_0.4.0               
##  [5] BiocFileCache_2.4.0         dbplyr_2.2.1               
##  [7] org.Mm.eg.db_3.15.0         AnnotationDbi_1.58.0       
##  [9] PoiClaClu_1.0.2.1           RColorBrewer_1.1-3         
## [11] pheatmap_1.0.12             forcats_0.5.2              
## [13] stringr_1.4.1               dplyr_1.0.10               
## [15] purrr_0.3.5                 readr_2.1.3                
## [17] tidyr_1.2.1                 tibble_3.1.8               
## [19] ggplot2_3.4.0               tidyverse_1.3.2            
## [21] DESeq2_1.36.0               SummarizedExperiment_1.26.1
## [23] Biobase_2.56.0              MatrixGenerics_1.8.1       
## [25] matrixStats_0.62.0          GenomicRanges_1.48.0       
## [27] GenomeInfoDb_1.32.4         IRanges_2.30.1             
## [29] S4Vectors_0.34.0            BiocGenerics_0.42.0        
## [31] tximeta_1.14.1             
## 
## loaded via a namespace (and not attached):
##   [1] readxl_1.4.1                  backports_1.4.1              
##   [3] AnnotationHub_3.4.0           systemfonts_1.0.4            
##   [5] lazyeval_0.2.2                splines_4.2.2                
##   [7] BiocParallel_1.30.4           digest_0.6.30                
##   [9] ensembldb_2.20.2              htmltools_0.5.3              
##  [11] fansi_1.0.3                   magrittr_2.0.3               
##  [13] memoise_2.0.1                 googlesheets4_1.0.1          
##  [15] tzdb_0.3.0                    Biostrings_2.64.1            
##  [17] annotate_1.74.0               modelr_0.1.9                 
##  [19] vroom_1.6.0                   timechange_0.1.1             
##  [21] prettyunits_1.1.1             colorspace_2.0-3             
##  [23] blob_1.2.3                    rvest_1.0.3                  
##  [25] rappdirs_0.3.3                textshaping_0.3.6            
##  [27] haven_2.5.1                   xfun_0.34                    
##  [29] crayon_1.5.2                  RCurl_1.98-1.9               
##  [31] jsonlite_1.8.3                tximport_1.24.0              
##  [33] genefilter_1.78.0             survival_3.4-0               
##  [35] glue_1.6.2                    gtable_0.3.1                 
##  [37] gargle_1.2.1                  zlibbioc_1.42.0              
##  [39] XVector_0.36.0                DelayedArray_0.22.0          
##  [41] car_3.1-1                     abind_1.4-5                  
##  [43] scales_1.2.1                  DBI_1.1.3                    
##  [45] rstatix_0.7.1                 Rcpp_1.0.9                   
##  [47] xtable_1.8-4                  progress_1.2.2               
##  [49] bit_4.0.4                     httr_1.4.4                   
##  [51] ellipsis_0.3.2                farver_2.1.1                 
##  [53] pkgconfig_2.0.3               XML_3.99-0.12                
##  [55] sass_0.4.2                    locfit_1.5-9.6               
##  [57] utf8_1.2.2                    labeling_0.4.2               
##  [59] tidyselect_1.2.0              rlang_1.0.6                  
##  [61] later_1.3.0                   munsell_0.5.0                
##  [63] BiocVersion_3.15.2            cellranger_1.1.0             
##  [65] tools_4.2.2                   cachem_1.0.6                 
##  [67] cli_3.4.1                     generics_0.1.3               
##  [69] RSQLite_2.2.18                broom_1.0.1                  
##  [71] evaluate_0.18                 fastmap_1.1.0                
##  [73] ragg_1.2.4                    yaml_2.3.6                   
##  [75] knitr_1.40                    bit64_4.0.5                  
##  [77] fs_1.5.2                      KEGGREST_1.36.3              
##  [79] AnnotationFilter_1.20.0       nlme_3.1-160                 
##  [81] mime_0.12                     xml2_1.3.3                   
##  [83] biomaRt_2.52.0                compiler_4.2.2               
##  [85] rstudioapi_0.14               filelock_1.0.2               
##  [87] curl_4.3.3                    png_0.1-7                    
##  [89] interactiveDisplayBase_1.34.0 ggsignif_0.6.4               
##  [91] reprex_2.0.2                  geneplotter_1.74.0           
##  [93] bslib_0.4.1                   stringi_1.7.8                
##  [95] highr_0.9                     GenomicFeatures_1.48.4       
##  [97] lattice_0.20-45               ProtGenerics_1.28.0          
##  [99] Matrix_1.5-1                  vctrs_0.5.0                  
## [101] pillar_1.8.1                  lifecycle_1.0.3              
## [103] BiocManager_1.30.19           jquerylib_0.1.4              
## [105] bitops_1.0-7                  httpuv_1.6.6                 
## [107] rtracklayer_1.56.1            R6_2.5.1                     
## [109] BiocIO_1.6.0                  promises_1.2.0.1             
## [111] codetools_0.2-18              assertthat_0.2.1             
## [113] rjson_0.2.21                  withr_2.5.0                  
## [115] GenomicAlignments_1.32.1      Rsamtools_2.12.0             
## [117] GenomeInfoDbData_1.2.8        mgcv_1.8-41                  
## [119] parallel_4.2.2                hms_1.1.2                    
## [121] grid_4.2.2                    rmarkdown_2.18               
## [123] carData_3.0-5                 googledrive_2.0.0            
## [125] shiny_1.7.3                   lubridate_1.9.0              
## [127] restfulr_0.0.15
```
