# Supplementary material for: Transcriptional coactivation by EHMT2 restricts glucocorticoid-induced insulin resistance in a study with male mice
Source: Nat Commun. 2023 May 30;14:3143. doi: 10.1038/s41467-023-38584-5 (PMC10229547; doi:10.1038/s41467-023-38584-5)
Supplement: Supplementary file 6 — Reporting Summary [file 41467_2023_38584_MOESM6_ESM.pdf]

nature portfolio

Corresponding author(s): Jen-Chywan Wang, PhD

Last updated by author(s): 04/07/2023

Reporting Summary

Nature Portfolio wishes to improve the reproducibility of the work that we publish. This form provides structure for consistency and transparency in reporting. For further information on Nature Portfolio policies, see our Editorial Policies and the Editorial Policy Checklist.

Statistics

For all statistical analyses, confirm that the following items are present in the figure legend, table legend, main text, or Methods section.

n/a	Confirmed	
☐	☒	The exact sample size (*n*) for each experimental group/condition, given as a discrete number and unit of measurement
☐	☒	A statement on whether measurements were taken from distinct samples or whether the same sample was measured repeatedly
☐	☒	The statistical test(s) used AND whether they are one- or two-sided<br>*Only common tests should be described solely by name; describe more complex techniques in the Methods section.*
☒	☐	A description of all covariates tested
☐	☒	A description of any assumptions or corrections, such as tests of normality and adjustment for multiple comparisons
☐	☒	A full description of the statistical parameters including central tendency (e.g. means) or other basic estimates (e.g. regression coefficient) AND variation (e.g. standard deviation) or associated estimates of uncertainty (e.g. confidence intervals)
☐	☒	For null hypothesis testing, the test statistic (e.g. *F*, *t*, *r*) with confidence intervals, effect sizes, degrees of freedom and *P* value noted<br>*Give P values as exact values whenever suitable.*
☒	☐	For Bayesian analysis, information on the choice of priors and Markov chain Monte Carlo settings
☒	☐	For hierarchical and complex designs, identification of the appropriate level for tests and full reporting of outcomes
☒	☐	Estimates of effect sizes (e.g. Cohen's *d*, Pearson's *r*), indicating how they were calculated

Our web collection on statistics for biologists contains articles on many of the points above.

Software and code

Policy information about availability of computer code

Data collection	Data was collected in Excel version 16.71. For RNA seq: Reads from BGI were first trimmed to remove adapters (TrimGalore! v0.6.6: trim_galore –gzip sample_1.fastq.gz) , then mapped to the ensemble transcriptome (Mus_musculus.GRCm38.cdna.all.fa.gz) using Salmon (v1.3.0: salmon quant -i GRCm38_index -l A -r sample_1_trimmed.fq.gz -p 4 --validateMappings --seqBias --gcBias --numBootstraps 50 -o quants/sample_1_quant). Count tables were imported into R using tximeta (v 1.9.3) using the gene model Mus_musculus.GRCm38.101.gtf.gz.
Data analysis	Data was analyzed in Prism version 9 software (Graphpad). For RNA seq: Differential expression performed using DESeq2 (v 1.30.1, and fit to the model ~dex + mutant + dex:mutant). A more detailed accounting of the processing in R is included as an Rmarkdown file in the Supplemental File 1

For manuscripts utilizing custom algorithms or software that are central to the research but not yet described in published literature, software must be made available to editors and reviewers. We strongly encourage code deposition in a community repository (e.g. GitHub). See the Nature Portfolio guidelines for submitting code & software for further information.

Data

Policy information about availability of data

All manuscripts must include a data availability statement. This statement should provide the following information, where applicable:
- Accession codes, unique identifiers, or web links for publicly available datasets
- A description of any restrictions on data availability
- For clinical datasets or third party data, please ensure that the statement adheres to our policy

The RNA-seq data generated in this study have been deposited in the NCBI Gene Expression Omnibus database under accession code GSE179180 [https://www.ncbi.nlm.nih.gov/geo/query/acc.cgi?acc=GSE179180]. GCRm38 was used for mapping.

Human research participants

Policy information about studies involving human research participants and Sex and Gender in Research.

Reporting on sex and gender	N/A
Population characteristics	N/A
Recruitment	N/A
Ethics oversight	N/A

Note that full information on the approval of the study protocol must also be provided in the manuscript.

Field-specific reporting

Please select the one below that is the best fit for your research. If you are not sure, read the appropriate sections before making your selection.

☒ Life sciences ☐ Behavioural & social sciences ☐ Ecological, evolutionary & environmental sciences

For a reference copy of the document with all sections, see nature.com/documents/nr-reporting-summary-flat.pdf

Life sciences study design

All studies must disclose on these points even when the disclosure is negative.

Sample size	The sample size is decided based on the statistics and our prior experience (Science Signaling paper). We use a Statistics Program (http://www.stat.ubc.ca/~rollin/stats/ssize/n2.html) to calculate the sample size. The power is set at 95% and α at 0.05. If the difference between two groups is bigger or equivalent to 30%, and standard deviation (SD) is 15%, the calculated minimum number is seven. Based on our experience, a minimum of six mice per treatment group are sufficient to achieve statistical power to detect the significant differences. For some studies using transgenic animals, we were limited by the numbers of the animals available for the experiments during the time we performed the studies. All group numbers are reported in the figure legends and source data.
Data exclusions	No data were excluded from analysis
Replication	Experiments were repeated independently 2-3 times and were successfully reproducible
Randomization	Mice in each cohort were either cohoused or individually housed and each cage was randomly assigned to a treatment group.
Blinding	For the experiments that were performed by a single person, the blinding was difficult. This was especially the case in the animal experiments, as the researchers already knew the treatment groups of mice. For some experiments, they were conducted blind. In this case, undergraduate assistants in the lab performed the experiments for the samples that were coded by number and they did not know the exact treatments of the samples until they finished the experiments.

Reporting for specific materials, systems and methods

We require information from authors about some types of materials, experimental systems and methods used in many studies. Here, indicate whether each material, system or method listed is relevant to your study. If you are not sure if a list item applies to your research, read the appropriate section before selecting a response.

Materials & experimental systems

n/a	Involved in the study
☐	☒ Antibodies
☒	☐ Eukaryotic cell lines
☒	☐ Palaeontology and archaeology
☐	☒ Animals and other organisms
☒	☐ Clinical data
☒	☐ Dual use research of concern

Methods

n/a	Involved in the study
☒	☐ ChIP-seq
☒	☐ Flow cytometry
☒	☐ MRI-based neuroimaging

Antibodies

Antibodies used

GAPDH (Proteintech, Rosemont, IL, 10494-1-AP)
EHMT2 (Sigma, St. Louis, MO, SAB2100657)
DUSP4 (Cell Signaling, Danvers, MA, 5149)
H3K9ME2 (Abcam, Cambridge UK, ab32521)
IRS2 (Cell Signaling, Danvers, MA, 3089S)
PCK1 (Proteintech, Rosemont, IL, 16754-1-AP)
G6PC (Proteintech, Rosemont, IL, 22169-1-AP)
Li-Cor goat anti rabbit (Li-Cor, Lincoln, NE, 926-32211)
Li-Cor goat anti mouse 800 (LiCor Lincoln, NE, 926-32210)
IgG (GeneScript, Piscataway, NJ, A01008)
GR (IA-1; a polyclonal rabbit antibody raised against human GR amino acids 84–112 QPDLSKAVSLSMGLYMGETETKVMGNDLG)
EHMT2 (Abcam, Cambridge, UK, 40542)
CBX3 (Abcam, Cambridge, UK, 10480)
H3K9ME2 (Abcam, Cambridge, UK, 32521)
Alpha Tubulin (Proteintech, Rosemont, IL, 11224-1-AP)

Validation

All antibodies have been validated according to the supplier's data sheets.
https://www.ptglab.com/products/GAPDH-Antibody-10494-1-AP.htm
https://www.sigmaaldrich.com/US/en/product/sigma/sab2100657
https://www.cellsignal.com/products/primary-antibodies/dusp4-mkp2-d9a5-rabbit-mab/5149
https://www.abcam.com/histone-h3-di-methyl-k9-antibody-y49-chip-grade-ab32521.html
https://www.cellsignal.com/products/primary-antibodies/irs-2-l1326-antibody/3089
https://www.ptglab.com/products/PCK1-Antibody-16754-1-AP.htm
https://www.ptglab.com/products/G6PC-Antibody-22169-1-AP.htm
https://www.licor.com/bio/reagents/irdye-800cw-goat-anti-rabbit-igg-secondary-antibody?utm_source=google&utm_medium=adwords&utm_content=reagent-webpage&utm_campaign=reagents&gclid=Cj0KCQiAsoycBhC6ARIsAPPbeLtTutVOgysKvGMZvsJiFlb8NKeqhMlKJpvdDnvGxajuyfrq1w20zrQaAgIOEALw_wcB
https://www.licor.com/bio/reagents/irdye-800cw-goat-anti-mouse-igg-secondary-antibody
https://www.genscript.com/antibody/A01008-Rabbit_IgG_Control_Whole_Molecule_Purified_.html
https://www.abcam.com/ehmt2g9a-antibody-ab40542.html
https://www.abcam.com/hp1-gammacbx3-antibody-ab10480.html
https://www.abcam.com/histone-h3-di-methyl-k9-antibody-y49-chip-grade-ab32521.html
https://www.ptglab.com/products/TUBA1B-Antibody-11224-1-AP.htm
We also validated and determined the appropriate concentrations before using in our experiments.

Animals and other research organisms

Policy information about studies involving animals; ARRIVE guidelines recommended for reporting animal research, and Sex and Gender in Research

Laboratory animals

For this study, C57BL/6J mice and EHMT2 mutant mice mice were used. EHMT2 mutant mice (Ehmt2K182R/K182R) were created with the help of The Washington University Genome Engineering and iPSC Center which performed in vitro validation of the gRNA needed for the CRISPR experiments in N2a cells. We chose gRNA (AGGTTTGGACATGGTTTTCCNGG) that not only mutated K182 to R (amino acid numbers are based on mouse Ehmt2 isoform b, NP_671493.1) but also had the least off target sites. Once the gRNA needed to create this mutation was identified in vitro, it was injected into zygotes (C57BL/6J) along with the Cas9 protein. The blastocysts derived from the injected zygotes were implanted into foster mice. These steps were carried out at the Gene Targeting Facility of the Cancer Research Institute at UC Berkeley. Overall, 9 founders were born, and genotyping showed that 8 of them had homozygous mutations that convert AAA to AGG. We bred these homozygous Ehmt2K182R/K182R mice for our preliminary studies. Mice were co-housed or individually housed in a temperature-controlled room of approximately 220C with 30-70% humidity in ventilated cages with a 12 hour light and dark cycle. Cages include Sanichip bedding along with a cotton Nestlet and a 4 g pick of crinkled paper. Mice were cohoused and were fed ad libitum a diet of PicoLab Rodent diet 5053 which contains 20% protein, crude fat 4.5%, and Fiber 6.0%. For the following experiments, randomly assigned male mice 8-12 weeks old were used. All experiments were approved by the University of California, Berkeley (AUP-2014-07-6617)

Wild animals

No wild animals were used

Reporting on sex	Male mice were used for the in vivo studies.
Field-collected samples	No field collected samples were used in this study
Ethics oversight	All experiments were approved by the Office of Laboratory Animal Care at the University of California, Berkeley (AUP-2014-07-6617)

Note that full information on the approval of the study protocol must also be provided in the manuscript.
